# Supplementary figures and images for: Myosin II mediates Shh signals to shape dental epithelia via control of cell adhesion and movement
Source: PLoS Genet. 2024 Jun 10;20(6):e1011326. doi: 10.1371/journal.pgen.1011326 (PMC11192418; doi:10.1371/journal.pgen.1011326)

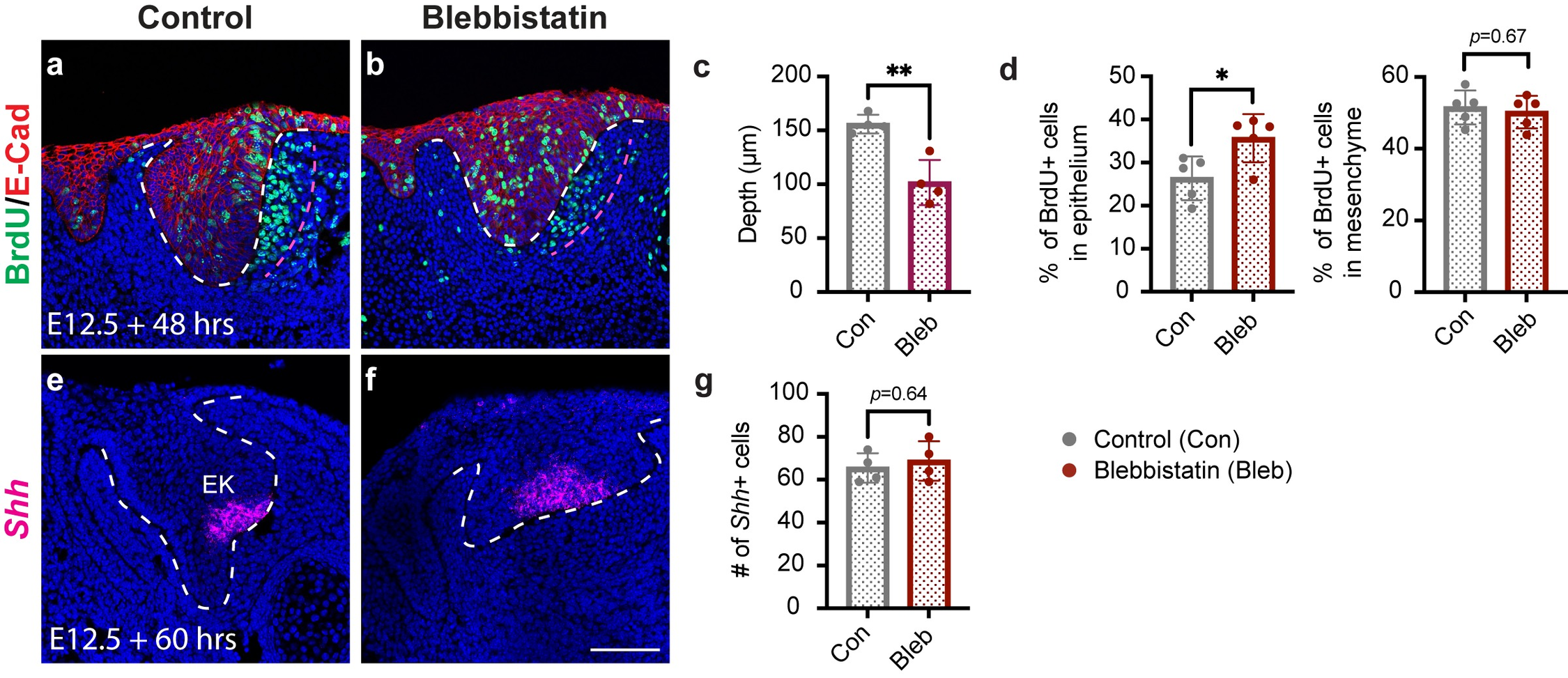

Supplement: S1 Fig — (a and b) BrdU labelling and E-Cad immunostaining on incisor sagittal sections of E12.5 mandible explants cultured in DMSO (control) or blebbistatin for 48 hours; anterior to the left (a and b). (c) Quantification of the epithelial depth in control and blebbistatin-treated incisors. (n = 4 embryos for each group). (d) Quantifications of the percentage of BrdU-positive (+) cells per section in control and blebbistatin-treated incisor epithelia (left panel) and posterior mesenchyme (right panel) (n = 5 embryos for each group). The posterior mesenchyme encompasses the area that is outlined by the pink dashed lines in (a and b). (e and f) RNAscope In situ hybridization of Shh shows normal formation of the enamel knot in both control and blebbistatin-treated samples after 60 hours of culturing. (g) Quantification of the number of Shh+ cells in control and blebbistatin-treated incisors. (n = 4 embryos for each group). White dashed lines outline the incisor epithelium. Representative images are shown. All quantitative data are presented as mean ± SD. The p values were determined using unpaired Student’s t-test for c, d, and g. (* p < 0.05, ** p < 0.01). Scale bar in (f) represents 50 μm in (a, b) and 80 μm in (e,f). (TIF) [file pgen.1011326.s001.tif]

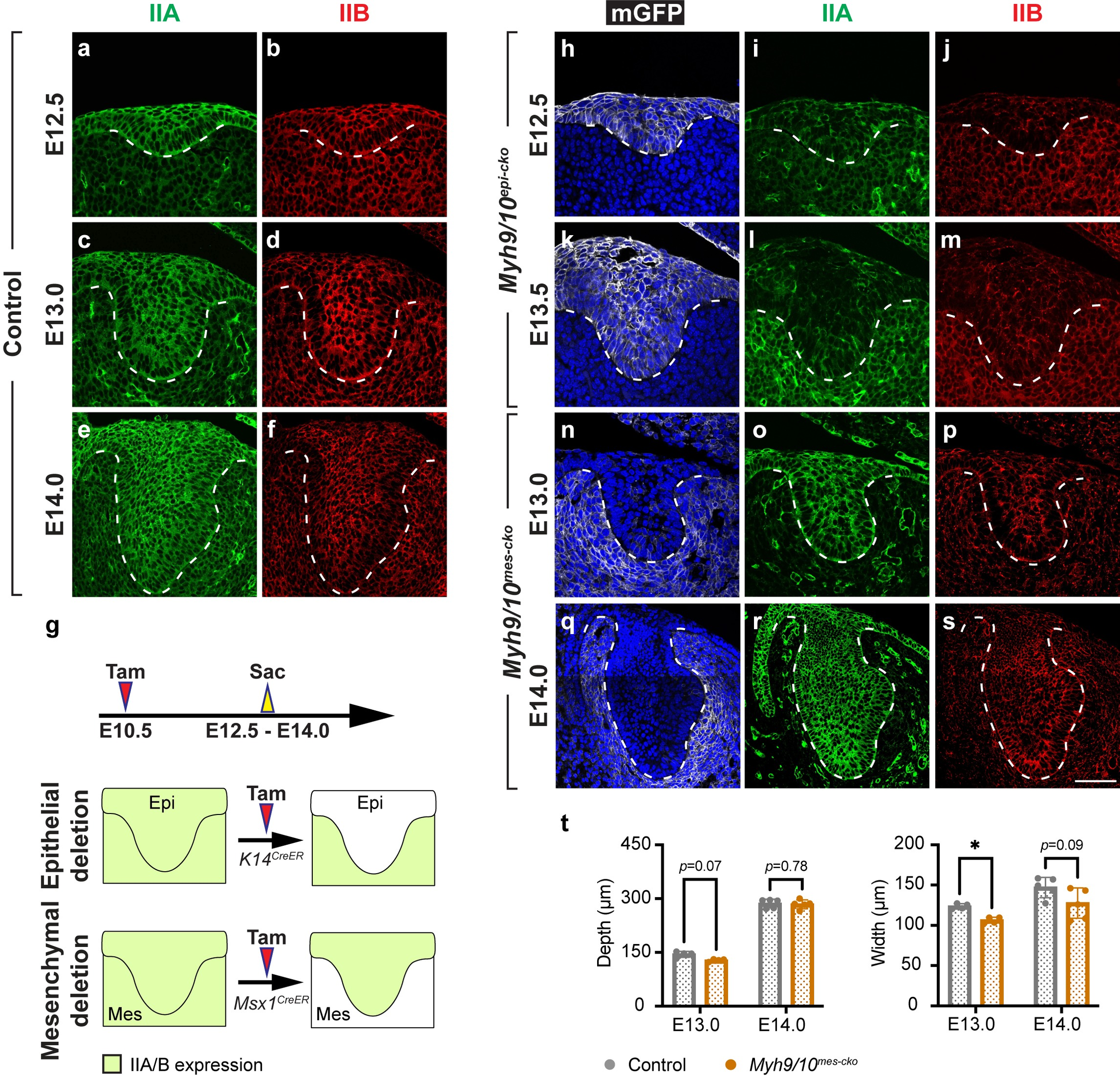

Supplement: S2 Fig — (a-f) Expression of myosin IIA and IIB in control incisor germs from E12.5 to E14.0. (g) Timeline depicting the onset of CreER induction by tamoxifen (Tam) through oral gavage (red arrowhead) and sample collection (yellow arrowhead). Bottom panel shows schematics of epithelial and mesenchymal deletion of IIA and IIB using K14CreER and Msx1CreER, respectively. (h-m) mGFP is a Cre-reporter, indicating K14-Cre mediated recombination in the Myh9/10epi-cko epithelium. Expression of myosin IIA and IIB are correspondingly reduced in the Myh9/10epi-cko dental epithelium at E12.5 and E13.5. (n-s) mGFP reporter shows Msx1-Cre activity in the dental mesenchyme. Myosin IIA and IIB expression are reduced in the Myh9/10mes-cko mesenchyme at E13.0 and E14.0. (t) Quantifications of the epithelial depth and the neck width in control and Myh9/10mes-cko incisors at E13.0 (n = 4 embryos for each group) and E14.0 (n = 5 embryos for each group). Dashed lines outline the incisor epithelium. Representative images are shown. All quantitative data are presented as mean ± SD. The p values were determined using unpaired Student’s t-test (* p < 0.05). Scale bar in (s) represents 50 μm in (a-d, h-p), 65 μm in (e and f), and 75 μm in (q-s). (TIF) [file pgen.1011326.s002.tif]

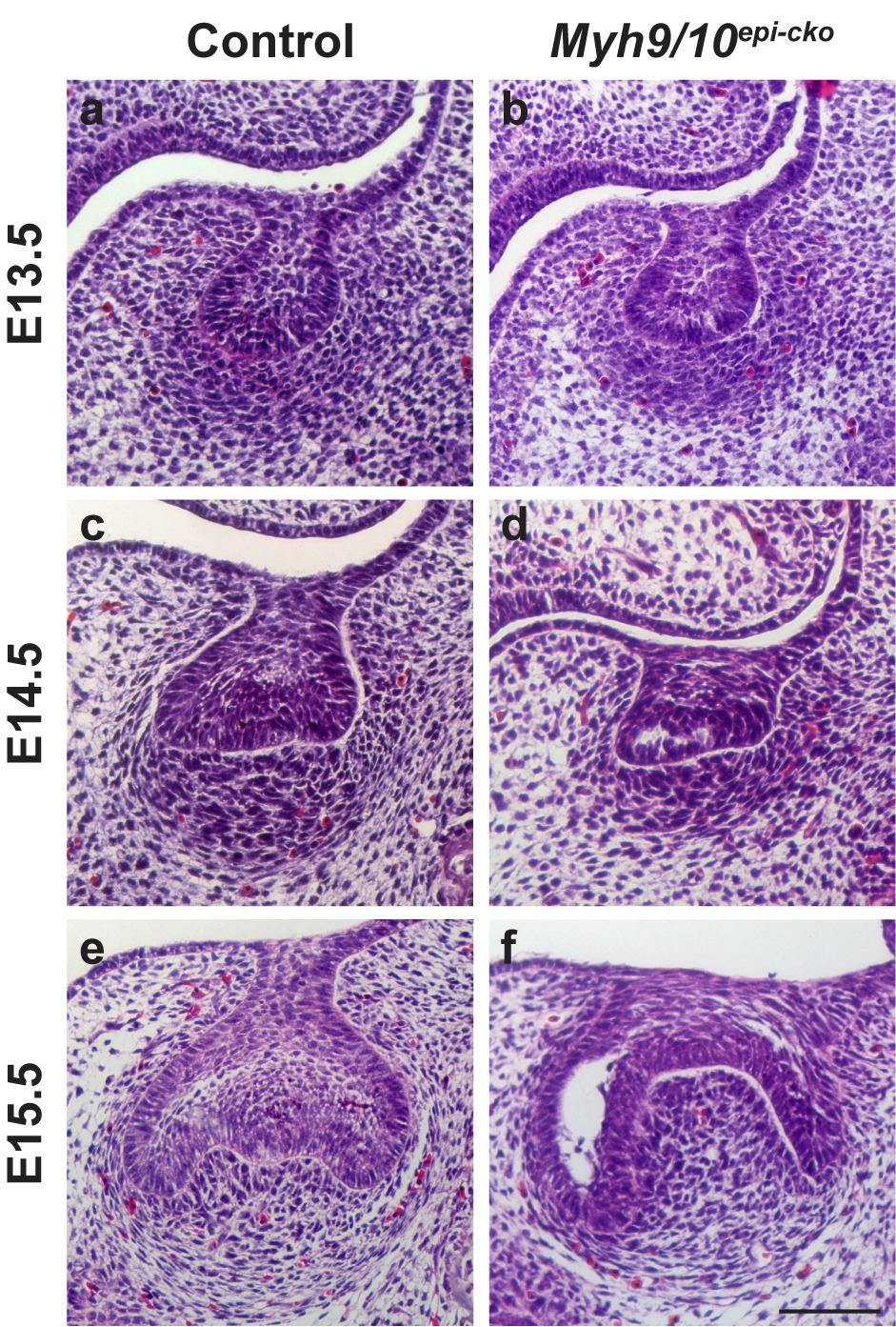

Supplement: S3 Fig — (a-f) H&E staining of E13.5-E15.5 control and Myh9/10epi-cko molar frontal sections. Lingual to the left and buccal to the right. Representative images are shown (n = 3 controls and 3 mutants). Scale bar in (f) represents 50 μm in (a-f). (TIF) [file pgen.1011326.s003.tif]

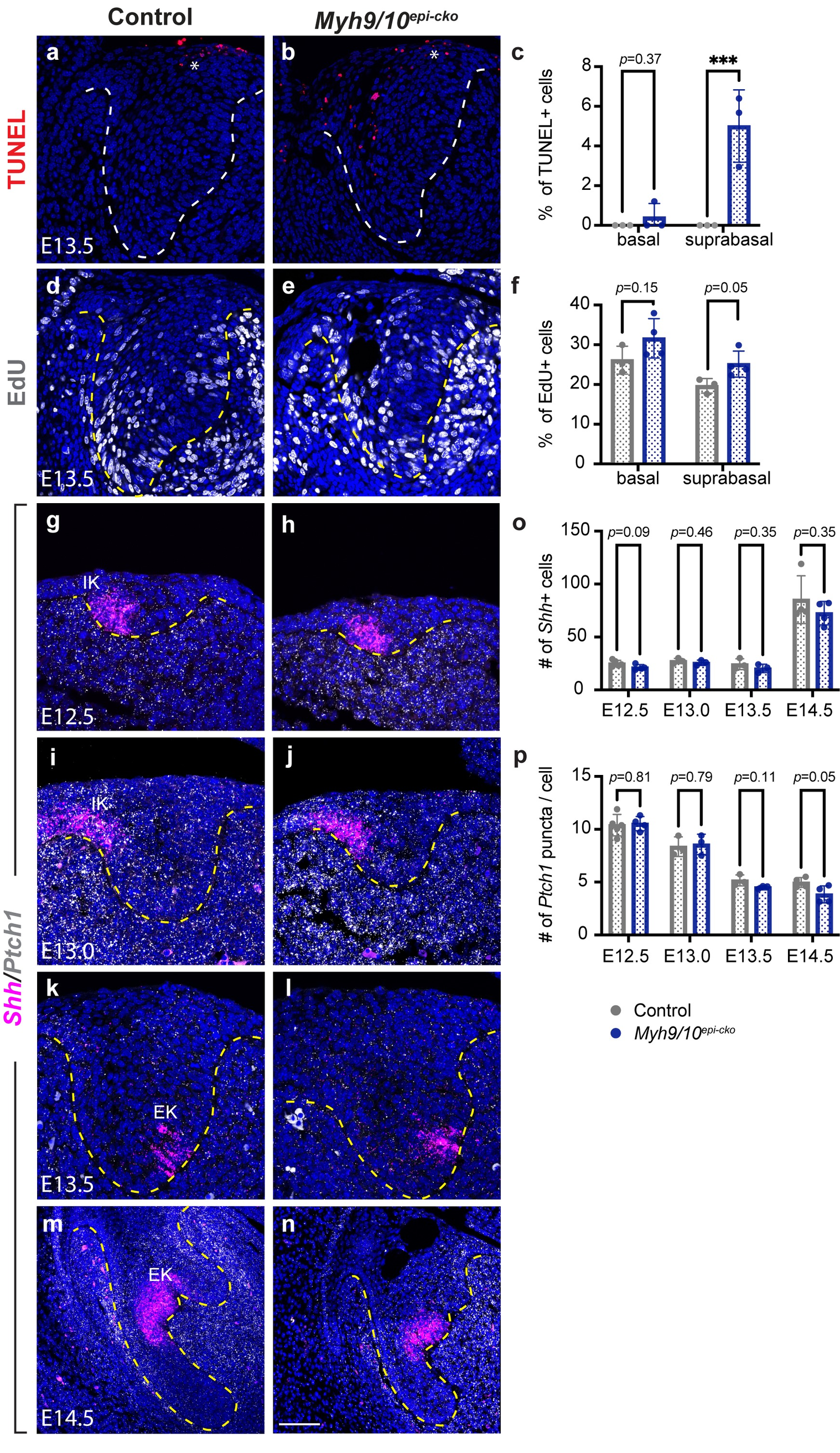

Supplement: S4 Fig — (a-c) TUNEL staining in control (a) and Myh9/10epi-cko (b) incisors shows increased cell death in suprabasal cells near the forming cyst upon Myh9/10 deletion (c). (n = 3 embryos for each group). Asterisks mark apoptosis in the superficial cells that is typically observed. (d-f) EdU labelling in control (d) and Myh9/10epi-cko (e) incisors shows a slight, albeit statistically insignificant, increase in cell proliferation upon Myh9/10 deletion (f). (n = 3 controls and 4 mutants). (g-p) RNAscope in situ hybridization of Shh and Ptch1 shows normal formation of the initiation knot (IK) and the enamel knot (EK), as well as normal Hedgehog signaling activity in the mutant incisor from E12.5 to E14.5 (g-n). Quantifications of the number of Shh+ cells and Ptch1 puncta per cell in control and Myh9/10epi-cko incisors at E12.5 (n = 5 controls and 4 mutants), E13.0 (n = 3 controls and 3 mutants), E13.5 (n = 3 controls and 3 mutants), and E14.0 (n = 4 controls and 4 mutants). White and yellow dashed lines outline the incisor epithelium. Representative images are shown. All quantitative data are presented as mean ± SD. The p values were determined using unpaired Student’s t-test for c, f, o, and p. (*** p < 0.001). Scale bar in (n) represents 65 μm in (a, b, d, e), 50 μm in (g, h, i, j, k, l), and 90 μm in (m and n). (TIF) [file pgen.1011326.s004.tif]

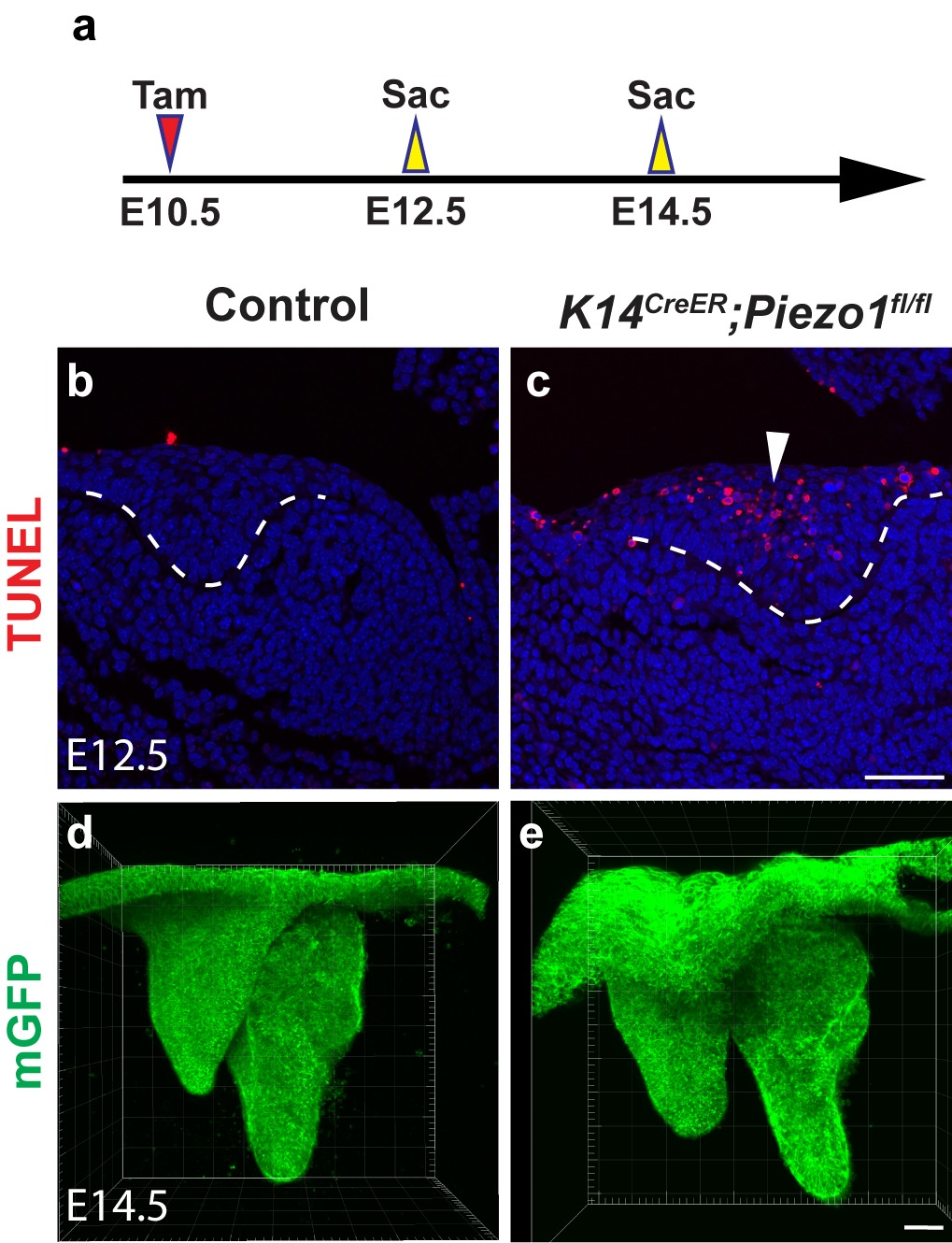

Supplement: S5 Fig — (a) Timeline depicting the onset of CreER induction by tamoxifen (Tam) through oral gavage (red arrowhead) to delete Piezo1 in the dental epithelium. Yellow arrowheads mark sample collections. (b-e) While deletion of Piezo1 resulted in increased cell death (white arrowhead) in the incisor bud at E12.5, as assessed by TUNEL staining (b,c), the mutant incisor was able to invaginate normally and no obvious morphological defects were observed at E14.5 under whole mount two-photon imaging (d and e). Dashed lines outline the incisor epithelium. Representative images are shown. Scale bar in (c) represents 50 μm in (b and c), scale bar in (e) represents 50 μm in (d and e). (TIF) [file pgen.1011326.s005.tif]

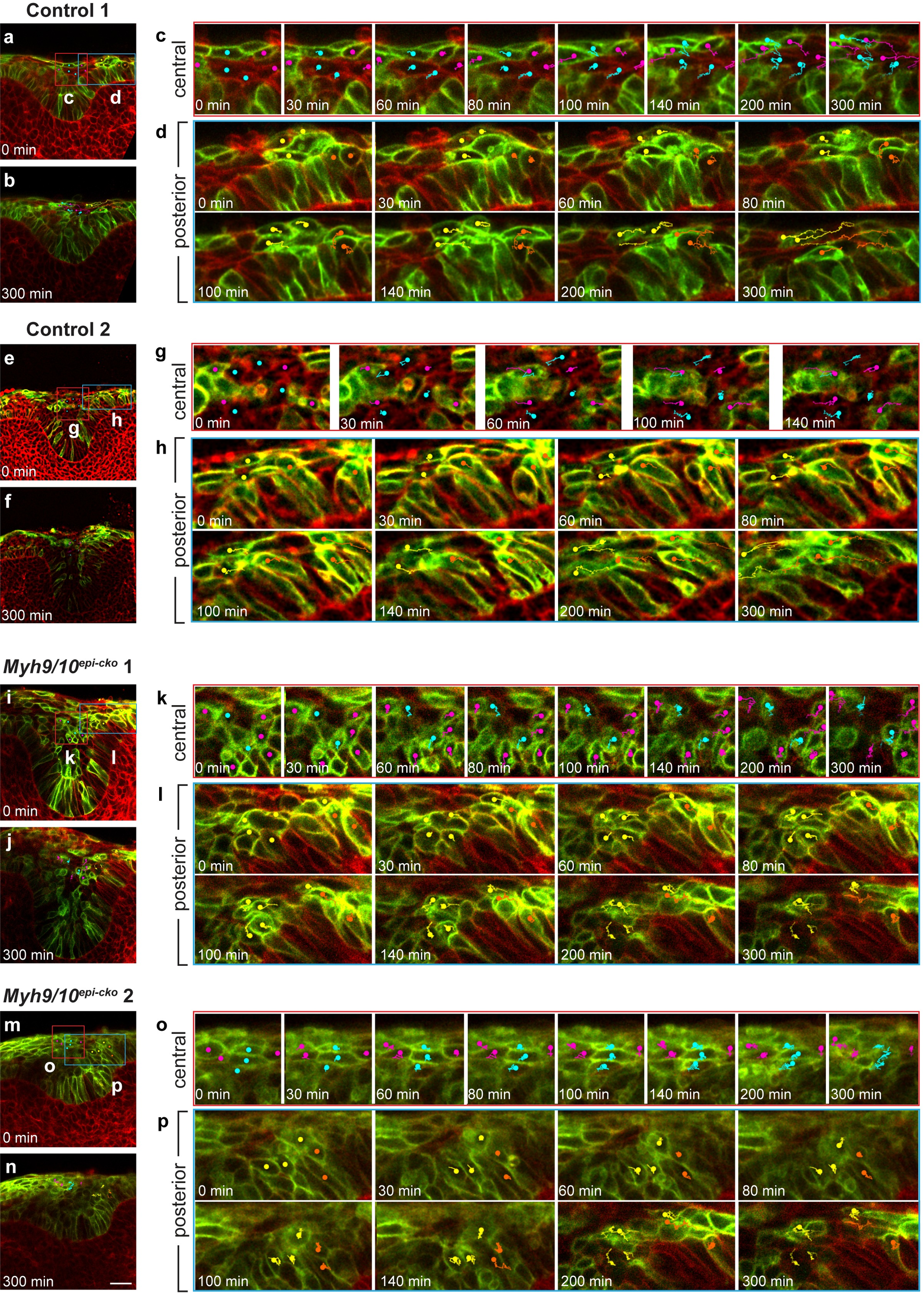

Supplement: S6 Fig — (a-p) Additional examples of two-photon time-lapse live imaging showing the tracked movement of E13.0 control (a-h) and mutant (i-p) cells from the central (red squares) and the posterior (cyan squares) regions of the upper incisor germ. Green dots mark anterior cells. Pink dots mark cells converging towards the midline. Cyan dots mark neighboring cells that are displaced by pink cells in the controls. Orange dots mark posterior basal cells and yellow dots mark adjacent co-migrating suprabasal cells. Cell membranes are labelled by the dual color CreER-reporter mTmG, through which CreER-mediated recombination induces the expression of membrane GFP, while red membrane tdTomato is expressed when there is no CreER activity. Representative images are shown. Scale bar in (n) represents 20 μm in (a, b, e, f, i, j, m, and n). (TIF) [file pgen.1011326.s006.tif]

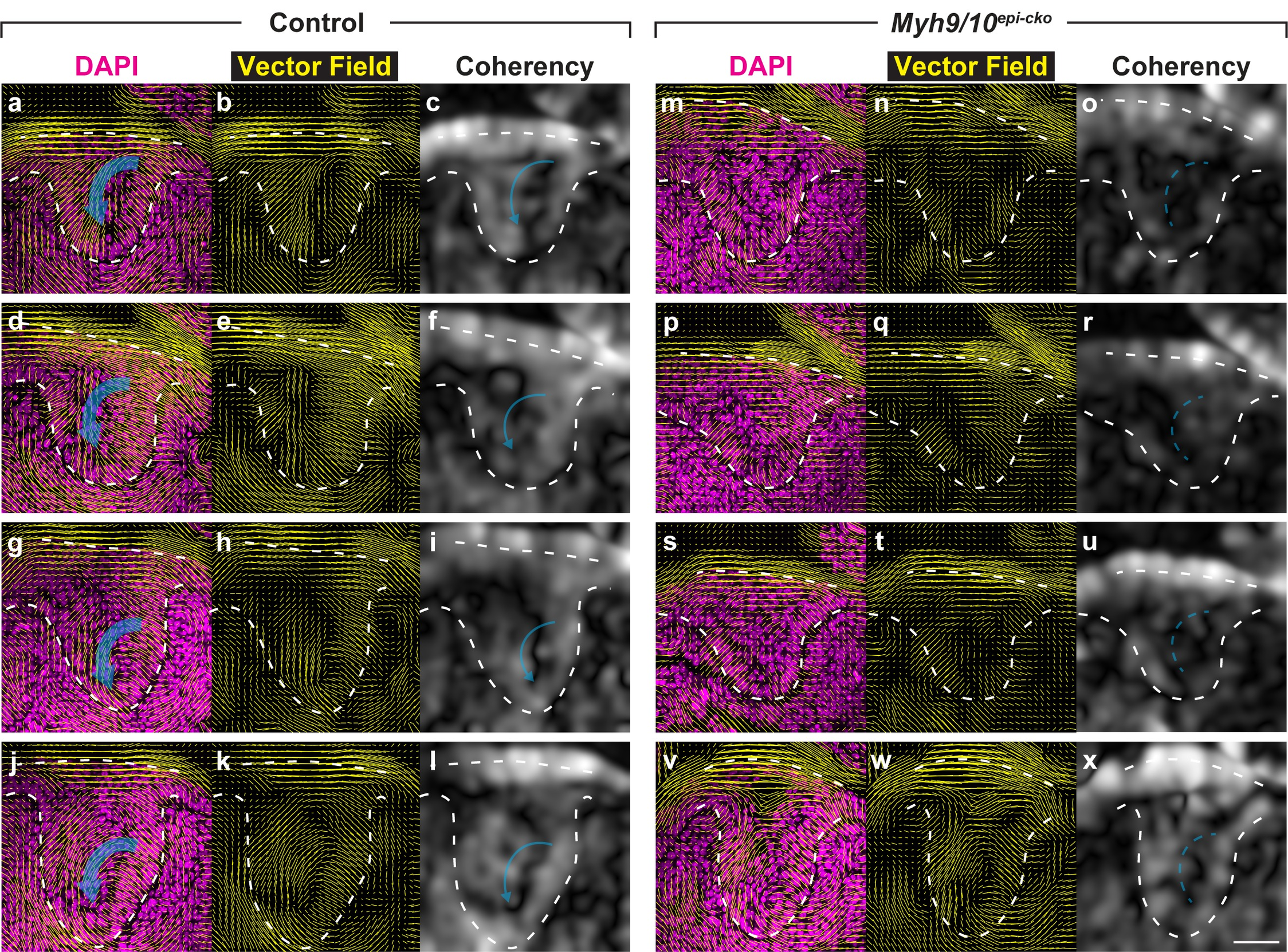

Supplement: S7 Fig — (a-l) Analysis of nuclear orientations in 4 representative E13.5 control incisor germs using OrientationJ reveals cell alignment around the forming enamel knot (blue arrows). Vector (yellow lines) orientations correspond to the averaged local nuclear orientations and vector length is proportional to the orientation coherency. Coherency maps show high orientation coherency in cells aligned around the enamel knot. (m-x) Compared to controls, E13.5 Myh9/10epi-cko mutant cells appear more disorganized. Cells around the enamel knot (dashed blue lines) are less coherent in their orientations and are less aligned around the enamel knot in vector fields. Dashed lines outline the incisor epithelium. Scale bar in (x) represents 50 μm in (a-x). (TIF) [file pgen.1011326.s007.tif]

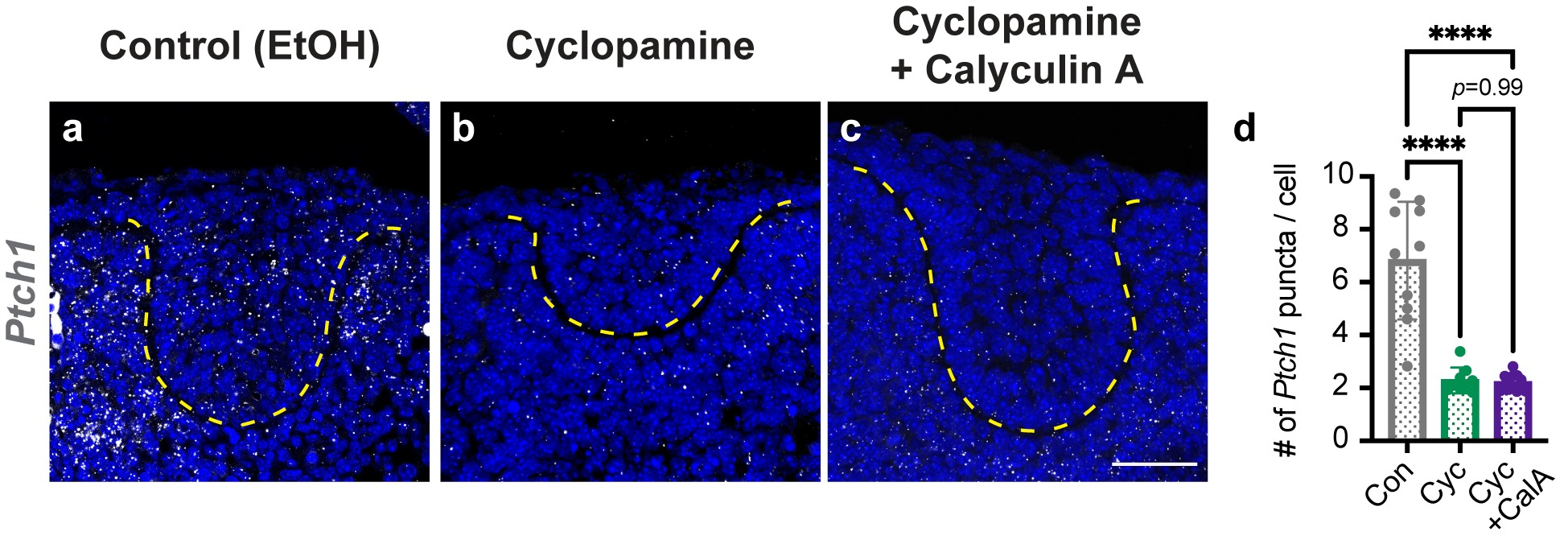

Supplement: S8 Fig — (a-c) RNAscope in situ hybridization of Ptch1 in control, Shh-inhibited (cyclopamine), and MyoII-rescued (cyclopamine plus calyculin A) incisors from E12.5 mandibles cultured for 24 hours. (d) Quantification of numbers of Ptch1 puncta per cell. (n = 10, 9, and 13 embryos respectively). Dashed lines outline the incisor epithelium. Representative images are shown. All quantitative data are presented as mean ± SD. The p values were determined using one-way ANOVA and Tukey’s HSD test. (**** p < 0.0001). Scale bar in (c) represents 50 μm in (a-c). (TIF) [file pgen.1011326.s008.tif]

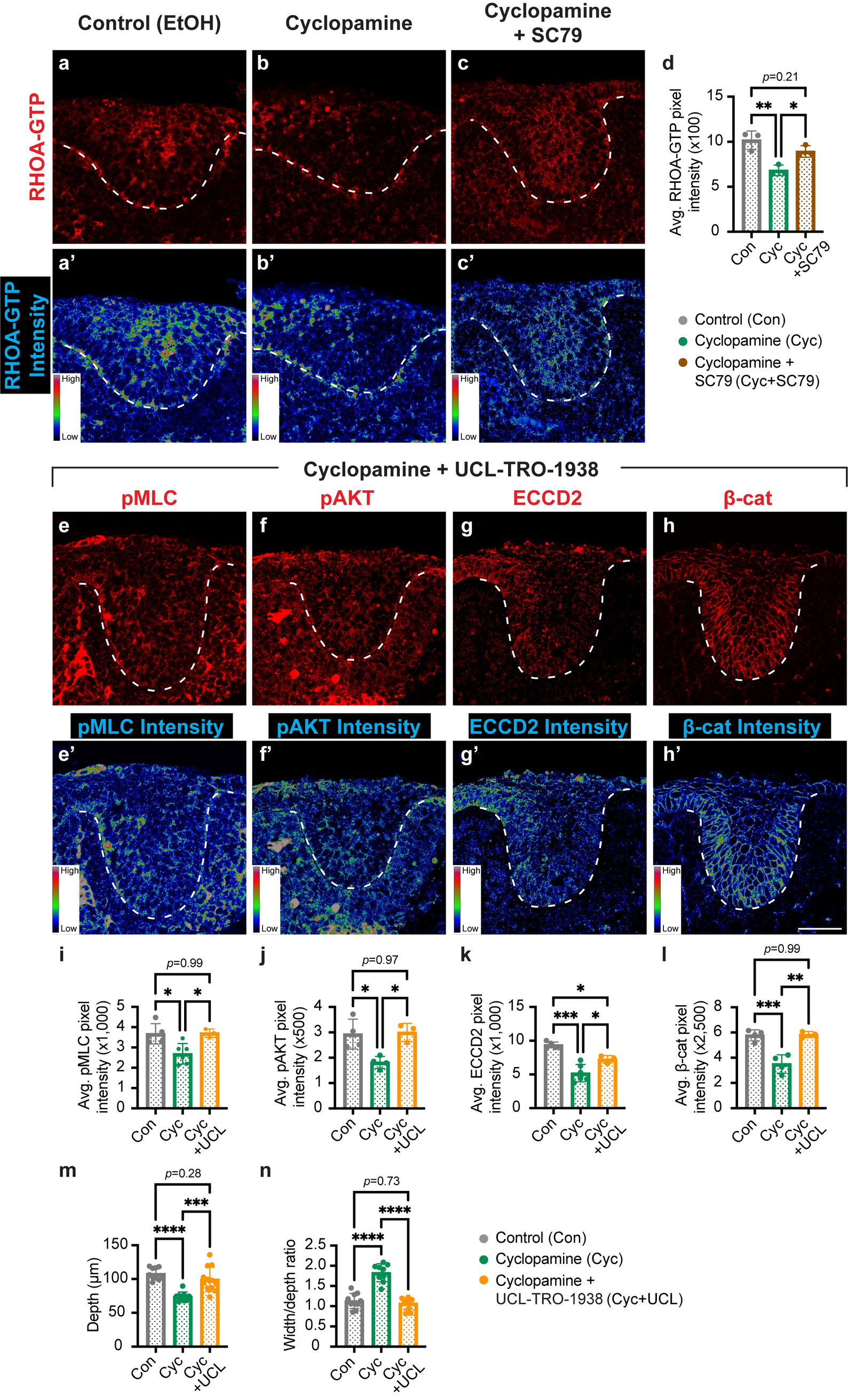

Supplement: S9 Fig — (a-d) Immunostaining of active RHOA (RHOA-GTP) (a-c) and corresponding signal intensity heatmaps (a’-c’) in control, Shh-inhibited (cyclopamine), and AKT-rescued (cyclopamine plus SC79) incisors from E12.5 mandibles cultured for 24 hours. The average RHOA-GTP signal intensity per pixel is quantified (d). (n = 3 for each group). (e-l) Immunostaining of pMLC (e), pAKT (f), ECCD2 (g), and β-catenin (β-cat) (h), as well as their corresponding signal intensity heatmaps (e’-h’) in PI3K-rescued (cyclopamine plus UCL-TRO-1938) incisors from E12.5 mandibles cultured for 24 hours. The average signal intensity per pixel for each stain is quantified (i-l). (n = 3 rescued samples for each stain. Data points for control and cyclopamine-treated samples are the same as Figures 6 and 7). (m and n) Quantifications of the incisor invagination depth (m), as well as the width to depth ratio (n) in control (n = 10), Shh-inhibited (cyclopamine, n = 9), and PI3K-rescued (cyclopamine plus UCL-TRO-1938, n = 12) samples. Dashed lines outline the incisor epithelium. Representative images are shown. All quantitative data are presented as mean ± SD. The p values were determined using one-way ANOVA and Tukey’s HSD test for d, and i-n. (* p < 0.05, ** p < 0.01, *** p < 0.001, **** p < 0.0001). Scale bar in (h’) represents 50 μm in (a-c’, e-h’). (TIF) [file pgen.1011326.s009.tif]

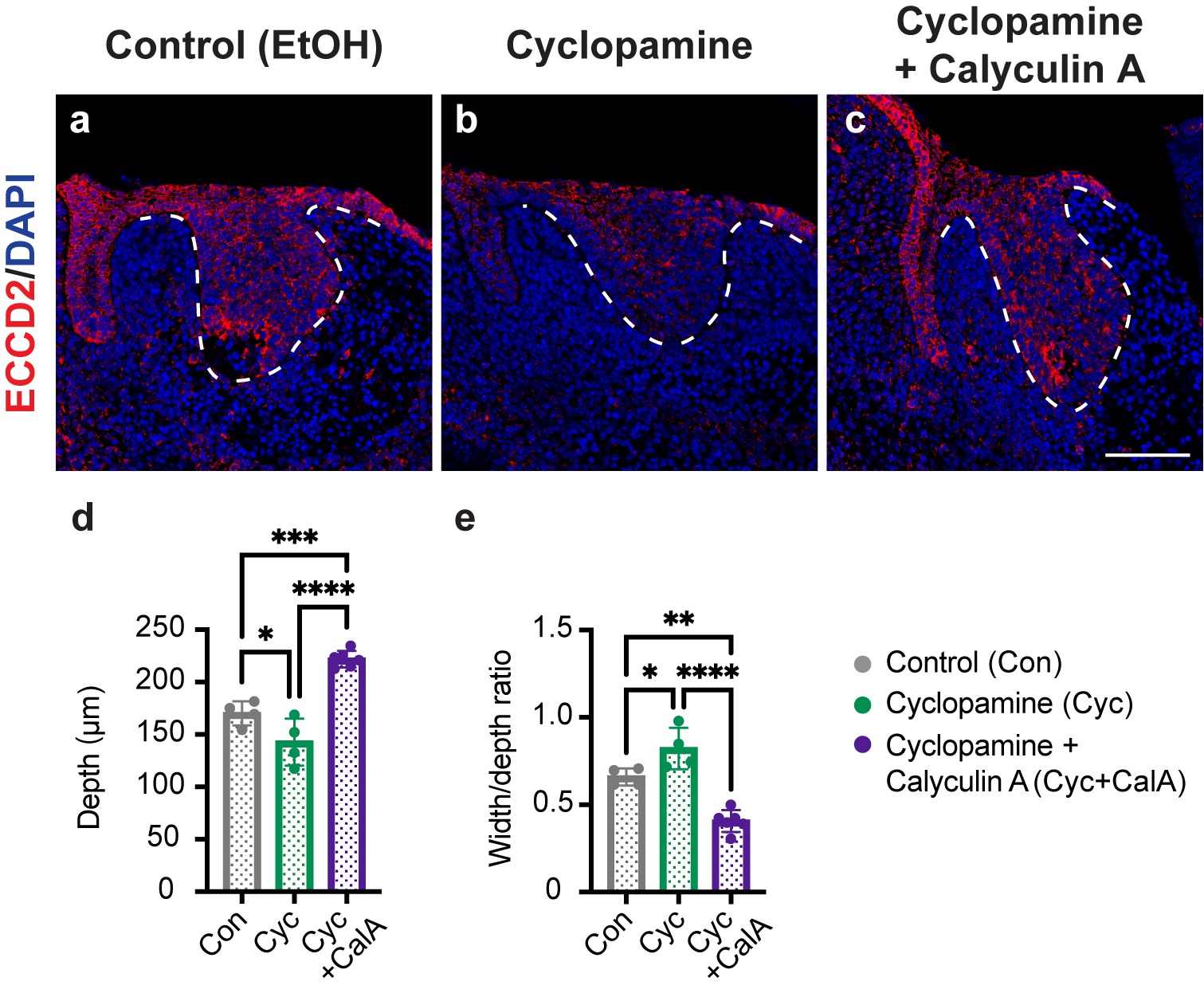

Supplement: S10 Fig — (a-c) Immunostaining of homophilically bound E-cad (ECCD2) in control, Shh-inhibited (cyclopamine), and MyoII-rescued (cyclopamine plus calyculin A) incisors from E13.5 mandible slices cultured for 24 hours. (d and e) Quantifications of the incisor invagination depth (d), as well as the width to depth ratio (e) in control (n = 4), Shh-inhibited (cyclopamine, n = 4), and MyoII-rescued (cyclopamine plus calyculin A, n = 6) samples. Dashed lines outline the incisor epithelium. Representative images are shown. All quantitative data are presented as mean ± SD. The p values were determined using one-way ANOVA and Tukey’s HSD test for d and e. (* p < 0.05, ** p < 0.01, *** p < 0.001, **** p < 0.0001). Scale bar in (c) represents 75 μm in (a-c). (TIF) [file pgen.1011326.s010.tif]

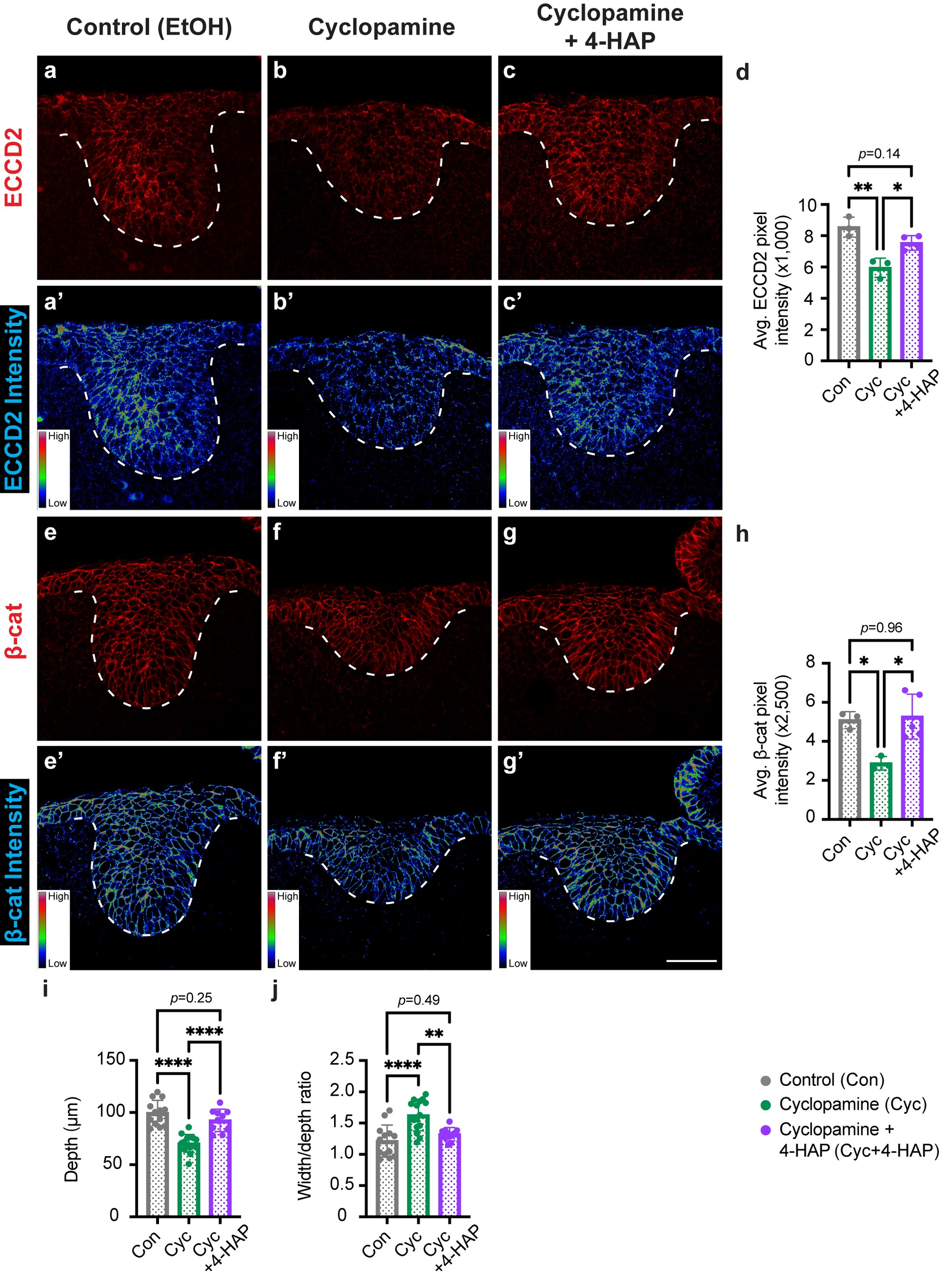

Supplement: S11 Fig — (a-d) Immunostaining of homophilically bound E-cad (ECCD2) (a-c) and corresponding signal intensity heatmaps (a’-c’) in control, Shh-inhibited (cyclopamine), and MyoII-rescued (cyclopamine plus 4-HAP) incisors from E12.5 mandibles cultured for 24 hours. The average ECCD2 signal intensity per pixel is quantified (d). (n = 3, 3, and 4 respectively). (e-h) Immunostaining of β-catenin (β-cat) (e-g) and corresponding signal intensity heatmaps (e’-g’) in control, Shh-inhibited (cyclopamine), and MyoII-rescued (cyclopamine plus 4-HAP) incisors from E12.5 mandibles cultured for 24 hours. The average β-cat signal intensity per pixel is quantified (h). (n = 3, 3, and 5 respectively). (I and j) Quantifications of the incisor invagination depth (i), as well as the width to depth ratio (j) in control (n = 13), Shh-inhibited (cyclopamine, n = 15), and MyoII-rescued (cyclopamine plus 4-HAP, n = 10) samples. Dashed lines outline the incisor epithelium. Representative images are shown. All quantitative data are presented as mean ± SD. The p values were determined using one-way ANOVA and Tukey’s HSD test for d, h, i and j. (* p < 0.05, ** p < 0.01, **** p < 0.0001). Scale bar in (g’) represents 50 μm in (a-c’, e-g’). (TIF) [file pgen.1011326.s011.tif]
