# Supplementary material for: Myosin II mediates Shh signals to shape dental epithelia via control of cell adhesion and movement
Source: PLoS Genet. 2024 Jun 10;20(6):e1011326. doi: 10.1371/journal.pgen.1011326 (PMC11192418; doi:10.1371/journal.pgen.1011326)
Supplement: Attachment — Submitted filename: Response to reviewers comments.pdf [file pgen.1011326.s024.pdf]

Reviewer #1: Manuscript Summary:

The topic of this study is of biological significance because it addresses an important question of how early tooth and ectodermal development occurs. The strength of this study partly lies in the innovative and well-established method of combining classical tissue explant culture and two-photon time-lapse microscopy, which allows the authors to precisely monitor individual cell movements. Previous studies have proposed a model similar to that in the current study, in which Shh promotes convergent cell migratory movement of the suprabasal tissue to drive dental epithelial invagination. However, the current study addresses the knowledge gap within the existing model for dental epithelial invagination by focusing on the role of non-muscle myosin II (MyoII), which is a key component of the actin-myosin filament interaction that forms contractile structures within the cells. The authors propose a mechanistic model, in which the Shh signaling is transmitted through MyoII to promote dental epithelial cell rearrangement. The model is based on: 1) functional study, both in vitro and in vivo, results in the MyoII-deficient incisor dental epithelium, showing that MyoII is essential for cell-cell adhesion and convergent cell movements in driving invagination; 2) rescue results in an in vitro Shh-deficient model combined with MyoII activation, showing that MyoII mediates the Shh function in dental epithelial invagination.

Response: We would like to first thank the reviewer for the positive feedback and for recognizing the importance of this study. The comments below are very helpful and by addressing them to the best of our ability, we believe that the manuscript is further improved.

Major Comments

1. When analyzing the Myh9/10 mutant mouse phenotype, they only showed the incisor. However, when discussing the underlying mechanisms, they mentioned other studies that described similar phenotypes, some of which were from studies done in the molar teeth. Thus, in the Myh9/10 mutant mice, could the authors additionally present the molar phenotypes and also discuss whether the defect phenotypes that they describe, including the "wider dental cord at the neck region" phenotype, were consistently found in both the incisor and molar?

Response: We thank the reviewer for the suggestion to also include the developing molars in this study. We have now generated frontal sections of *Myh9/10* mutant molar germs from E13.5 to E15.5 and observed that mutant molars too retained a wider dental cord and formed holes within the epithelial tissue. These phenotypes are consistent with what we observed in the developing incisors, and the representative H&E images are now added as Supplementary Figure 3 (S3 Fig). The following sentence, "*We also assessed the developing Myh9/10$^{epi\text{-}cko}$ molars from E13.5 to E15.5 and observed the same phenotypes*", is also added after our phenotypic description of the E15.5 incisors on page 8 of the revised manuscript with tracked changes.

2. When analyzing the Myh9/10 mutant mouse phenotype, the authors ruled out ‘cell death’ as the main cause of the defective invagination phenotype based on the phenotype in K14CreER;Piezo 1 mutant mice, However, does it make sense to rule out ‘cell death’ when it is combined with ‘cyst formation’? Do Piezo 1 mutant mice also show BOTH cell death and cyst formation? Couldn’t there be a possibility that the cyst could interrupt either/both the narrowing of the dental cord or/and the convergent extension event, especially considering the possible physical cell-cell interactions between outer- and central-located cells, which was previously by the Green lab to contribute to the ‘telescoping’ and other cell movements that mediate invagination? Could the author consider discussing this point?

Response: This is an excellent point, and we should have been more careful in drawing conclusions from comparing the Piezo1 and MyoII mutant phenotypes. Indeed, as we have not observed cysts in *Piezo1* mutants, we cannot rule out a combined effect of cell death and cyst formation on dental cord narrowing and tooth invagination. We have modified our sentence in both the Results and Discussion sections to state that “cell death alone” is unlikely to be the main cause of the invagination defect. We also added the following sentence to the Discussion, “*It remains to be determined whether a combined effect from both cell death and cyst formation can impede tooth invagination. As cell-cell connections normally persist between adjacent basal and suprabasal cells during invagination (Panousopoulou and Green, 2016), losing such connections at a large scale due to the presence of a cyst could disrupt tissue force transmission in MyoII mutants and further add to the invagination phenotype after E13.5.*”, on page 17-18 of the revised manuscript with tracked changes.

The above discussion would thus integrate findings from the Green lab, as the Reviewer pointed out. However, as the functional requirement of the basal-suprabasal cell connections has yet to be determined, this remains an important open question, which we hope to address in the future. In the case of the MyoII mutants, because phenotypes related to cell adhesion and migration took place at E13.0, before cell death and cyst formation at or after E13.5, we believe that at least the initial manifestation of the mutant phenotypes is independent of the cyst. We have now better pointed out these temporal relations in the Discussion (under “Control of epithelial adhesion and invagination by myosin II” on page 18 of the revised manuscript with tracked changes). We hope this will help readers appreciate how disrupted adhesions in MyoII mutants could contribute to the invagination phenotype at different levels and timepoints: at the cell level, reduced adhesion at adherence junctions accompanied the initial cell convergent movement defect; and then at the tissue level, cyst formations could further compromise epithelial invagination at later stages.

1. Panousopoulou E, Green JBA. Invagination of Ectodermal Placodes Is Driven by Cell Intercalation-Mediated Contraction of the Suprabasal Tissue Canopy. PLoS Biol. 2016;14: e1002405. Doi:10.1371/journal.pbio.1002405

3. The authors propose that Shh signaling is transmitted through MyoII to promote dental epithelial cell rearrangement. Then, similar to the Myh9/10 mutant mice, is there anything known about whether Shh mutant mice also show cyst formation? Similarly, were there any previous reports about cyclopamine-treat mice showing cyst formation? And in the current study, did the authors observe cyst formation in their results from the Shh deficiency experiment?

Response: While we observed a decrease in cell adhesion in cyclopamine-treated tissues, based on reduced immunostaining signals of ECCD2 (recognizing homophilically bound E-cadherin) and membrane-localized β-catenin, we did not observe any obvious cyst formation. This may be due to insufficient culture time for the cyst to form or a less severe adhesion loss than that in the MyoII mutants. We have included the observation (in terms of cyst formation) and our reasoning in the newly expanded Results section on page 15 (bottom) and page 16 (top) of the tracked manuscript that investigates the Shh-PI3K/AKT-MyoII signaling axis (see below).

We have also performed literature searches to examine if cystic formation has been found in other tissues with Shh loss-of-function. Interestingly, formation of keratinized cysts was described in teeth with epithelial Shh deletion that were grown in kidney capsules (Dassule et al., 2000). However, the original article did not provide an image. (N.B. The Shh and MyoII mutants share other invagination phenotypes; as Shh mutant “epithelial bud was wider and it had not invaginated as deeply”). Perturbation of Shh signaling (both up- and down-regulation) has also been shown to cause cystic kidney diseases (Bay et al., 2018; Sheybani-Deloui et al., 2018), but the cellular mechanisms are not well understood. We are therefore uncertain if these cystic phenotypes are equivalent to what we have observed in the tooth, and we have chosen not to cite these findings in our current study. On the other hand, the importance of Shh in regulating cell-cell adhesion via cadherins is well documented. We have therefore cited some of these studies in the discussion (middle of page 20 of the revised manuscript with tracked changes) to highlight that aspect of Shh function.

1. Dassule HR, Lewis P, Bei M, Maas R, McMahon AP. Sonic hedgehog regulates growth and morphogenesis of the tooth. Development. 2000;127: 4775–4785. doi:10.1242/dev.127.22.4775
2. Bay, S. N., Long, A. B. and Caspary, T. (2018). Disruption of the ciliary GTPase Arl13b suppresses Sonic hedgehog overactivation and inhibits medulloblastoma formation. Proceedings of the National Academy of Sciences 115, 1570–1575.
3. Sheybani-Deloui, S., Chi, L., Staite, M. V., Cain, J. E., Nieman, B. J., Henkelman, R. M., Wainwright, B. J., Potter, S. S., Bagli, D. J., Lorenzo, A. J., et al. (2018). Activated Hedgehog-GLI Signaling Causes Congenital Ureteropelvic Junction Obstruction. J Am Soc Nephrol 29, 532–544.

4. The molecular mechanisms that link Shh deficiency and MyoII function seems missing. The authors did discuss the possibility of a mechanism similar to how “Shh transcriptionally induces

the expression of small GTPase RhoU to control cell-cell adhesion and rearrangement for proper epithelial morphogenesis" from a previous study on a different organ. Could the authors check some of these markers so that it becomes more clear whether their proposed mechanism is possibly underlying non-canonical Shh signaling or not. As it stands, Shh-to-MyoII seems a bit of a leap.

Response: We appreciate the suggestion and have performed a series of experiments to address this important point. We began by first acquiring additional evidence to further support the Shh-MyoII link, as it was questioned by another reviewer. To that end, we measured the expression of active RHOA-GTP by immunostaining and showed that it was downregulated in cyclopamine-treated samples (shown as new data in S9a,b,d Fig). We also performed a new set of rescue experiments to restore MyoII activity using 4-HAP, which activates MyoII through a different mechanism than calyculin A. We were able to obtain the same results showing successful rescues (shown as new data in S11 Fig). These findings thus further strengthen our original conclusion that Shh promotes MyoII activity to drive tooth invagination.

We next tested the possibility that Shh signals through a non-canonical pathway to regulate MyoII, as suggested by the reviewer. We first assessed RhoU expression but did not detect differences between control and cyclopamine-treated samples (n = 3, representative images are shown below). We did not include this result in the revised manuscript due to space constraint.

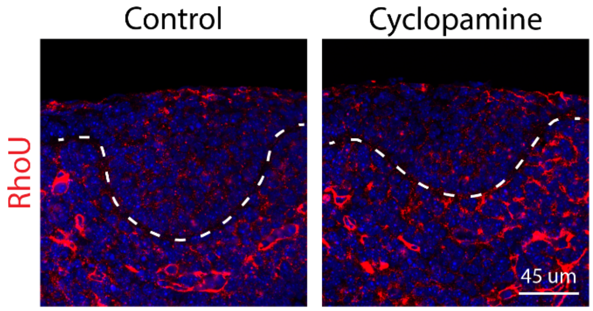

We then examined the activity of the PI3K/AKT pathway by performing pAKT immunostaining and found that to be significantly reduced in cyclopamine-treated samples (shown as new data in Fig. 6i,l). This prompted us to perform rescue experiments using pharmacological activators of AKT or PI3K, and we found that restoration of their activities not only rescued the expression of pMLC and active RHOA-GTP but also rescued the cell adhesion and migration phenotypes caused by cyclopamine treatment (shown as new data in Fig. 6f,k, Fig. 7d,i,n, and S9 Fig; texts on page 14-16 of the revised manuscript with tracked changes). These results thus indicate that Shh signals through PI3K/AKT to activate RHOA and MyoII in the developing incisor.

5. The in vitro experiment of the cyclopamine-treated incisors, rescue by calyculin treatment, suggests that MyoII mediates Shh function. Compared to all the solid results presented in this manuscript from their Myh9/10 mutant model, which nicely led up to this final 'rescue'

experiment, the evidences that support the ‘rescue’ lack evidence backed by their powerful live imaging experiment, which may truly pin down their proposed Shh-MyoII-cell rearrangement model. Was the live imaging done but did not yield positive results? Or if not done, I think it would be a great addition that complements the live imaging done in their Myh9/10 mutant mouse model.

Response: We too agree that identifying how cell movement is affected by cyclopamine and in rescue experiments will help elucidate the function of the Shh-MyoII signaling axis in promoting incisor invagination. We have therefore conducted a new set of live imaging experiments to track the movement of posterior dental epithelial cells in tissues treated with ethanol (control vehicle), cyclopamine, cyclopamine plus calyculin A (MyoII rescue) and cyclopamine plus SC79 (AKT rescue). As expected, cyclopamine-treated incisors could not effectively invaginate and their posterior cells failed to efficiently move towards the tooth midline, thus phenocopying Myh9/10 mutants. However, this defect was rescued when the activity of MyoII or AKT was restored. In these rescued samples, posterior cells exhibited a much-improved convergent movement efficiency that is statistically significant, when compared to cyclopamine-treated samples. Epithelial invagination could also be clearly observed in timelapse movies. These new data are now added as Fig. 7k-o and S8-11 Movies, with the accompanying texts on page 16 (top) of the revised manuscript with tracked changes. These data also complement well with our existing live imaging results from the Myh9/10 mutants, and together they strongly support a model in which the Shh-PI3K/AKT-MyoII signaling axis promotes cell convergent movement to drive dental epithelial invagination.

Minor Comment

1. “However, in the mutant incisor germs many of these foci were lost (Fig. 4I,j), suggesting a defect in cellular
interactions and convergence.”: “4I” should be uncapitalized.

Response: Thank you for catching this. We have corrected this mistake now.

Reviewer #2:

In this study, Du and colleagues examine the morphological mechanisms underlying the invagination of the dental placode. Using both a myosin II inhibitor and genetic loss of function experiments with Myosin II (Myosin 9/10 conditional knockouts specifically in the epithelium), they find that invagination is perturbed. Using live cell imagining, they show that directed cell migration is perturbed in conditional knockouts, indicating that Myosin II is required for normal convergent extension-type movements. Finally, using pharmacological approaches, they find that pharmacological inhibition of hedgehog signaling (cyclopamine) causes a similar phenotype to loss of Myosin II and that this can be rescued by co-administration of calyculin A. Based on

these findings they conclude that Hedgehog signaling mediates Myosin II signaling to regulate tooth morphogenesis.

I disagree with the overall conclusion of the study based on concerns with the interpretation of several experiments. In particular, the Myosin inhibition/loss-of-function experiments may actually be reducing the levels of Hedgehog signaling. If this is the case, then Hedgehog signaling is downstream consequence of myosin rather than upstream as suggested in the manuscript. This possibility is seemingly at odds with their conclusion that Hedgehog inhibition can be rescued by activating MLC. These experiments are based on the pharmacological rescue of the Hedgehog inhibitor cyclopamine by the phosphatase inhibitor calyculin A. But in addition to inhibiting MLC phosphatase, calyculin A has more broad-spectrum phosphatase activity that could interfere with other aspects of cell signaling and might even counteract the inhibitory effects of cyclopamine on Hedgehog signaling. If this scenario were to be true, it would mean that the 'rescue' is a trivial rescue of Hedgehog signaling from cyclopamine rather than a downstream activation of pMLC. An additional concern is with the experimental strategy of prolonged myosin loss (either 48 hours of blebistatin treatment or several days after Cre-mediated excision in the genetic model). Because Myosin II inhibition can have rather quick effects on cell behavior (within minutes or certainly a few hours), the observed phenotypes ay be far removed from the primary effect of myosin. As detailed below, these raise substantial concerns about the conclusions of this study.

Response: We thank the reviewer for bringing this very important point to our attention and for motivating us to look carefully at the possibility that Shh signaling can be influenced by MyoII and by calyculin A treatment. To address these questions, we have now (1) quantified *Shh* and *Ptch1* expression in MyoII mutants from E12.5 to E14.5; (2) quantified *Ptch1* expression in calyculin A-rescued samples; and (3) performed additional rescue experiments using 4-HAP, which activates MyoII through a different mechanism than calyculin A. In each case, we obtained results that are consistent with our initial conclusion that Hh signaling acts upstream of MyoII during early tooth development. We will further discuss these experiments and findings below. We do want to point out that there is perhaps a slight reduction in Shh signaling in the MyoII mutants at E14.5, but that reduction is not statistically significant. Nonetheless, in the Discussion we raised the possibility that MyoII may yet function to promote or propagate Shh signaling at later stages. The direction of the Shh-MyoII interaction does not need to be mutually exclusive, and our data provide evidences that support Shh's role as an upstream regulator of MyoII to narrow the dental cord and promote invagination.

Major Concerns:

1. Use of calyculin A in pharmacological rescue. In Drosophila, calyculin A or okadaic acid (another phosphatase inhibitor) cause changes in Smoothened mobility that cause its migration to resemble activated Smoothened forms seen in response to Hedgehog signaling (Denef et al, 2000 Cell 102:521-531). Other studies have suggested that PP1 and PP2a (both inhibited by calyculin A) can regulate Protein Kinase A substrates mediating Hedgehog signaling (reviewed

in Zhao et al 2017 Cell Commun Signal. Both of these suggest that calyculin A might be counteracting the inhibitory effects of cyclopamine on Hedgehog signaling itself (rather than just on activating pMLC), leading to a rescue of the phenotype. Examining Ptch1 expression and other HH pathway readouts in cyclopamine and cyclopamine plus calyculin A would help address this concern. In addition, independent ways of rescuing myosin activity would also be helpful.

Response: We completely agree with the reviewer that it will be important to demonstrate that calyculin A was not simply rescuing Shh signaling in our rescue experiments. As the reviewer pointed out, phosphorylation of Smo by a set of kinases, specifically PKA/CK1 in *Drosophila* and GRK2/CK1 in vertebrates, allows Smo to switch to an active conformation for signaling. But phosphorylation by CK1 can be countered by phosphatase PP2A, which is a target of calyculin A, hence the concern that calyculin A treatment can result in Smo re-activation. However, cyclopamine locks Smo in its closed conformation by blocking Smo phosphorylation at GRK/CK1 sites (Chen et al., 2011). Therefore, our expectation was that calyculin A would not be able to activate Smo in the presence of cyclopamine since phosphorylation on Smo is blocked. To verify this, we performed *Ptch1* RNAscope to examine its expression in samples treated with ethanol (control vehicle), cyclopamine, or cyclopamine plus calyculin A, as the reviewer suggested. The result showed that *Ptch1* expression remained low in calyculin A-treated samples (new data shown in S8 Fig with accompanying texts on page 15 (middle) of the revised manuscript with tracked changes), indicating that indeed calyculin A is not capable of bypassing cyclopamine-induced Smo inhibition to activate Shh signaling.

The reviewer also suggested that we could rescue MyoII activity using other methods. To that end, we performed a new set of rescue experiment using 4-hydroxyacetophenone (4-HAP) (Surcel et al., 2015), which increases actomyosin tension by promoting the assembly of myosin IIB and IIC without affecting their phosphorylation states. We obtained the same results as before, finding that 4-HAP was able to rescue the cell adhesion and invagination defects caused by cyclopamine (new data shown in S11 Fig). Together, these results add to our existing data and further demonstrate that restoration of MyoII activity rescues phenotypes triggered by Shh inhibition.

1. Chen, Y., Sasai, N., Ma, G., Yue, T., Jia, J., Briscoe, J. and Jiang, J. (2011). Sonic Hedgehog Dependent Phosphorylation by CK1α and GRK2 Is Required for Ciliary Accumulation and Activation of Smoothened. PLOS Biology 9, e1001083.
2. Surcel, A., Ng, W. P., West-Foyle, H., Zhu, Q., Ren, Y., Avery, L. B., Krenc, A. K., Meyers, D. J., Rock, R. S., Anders, R. A., et al. (2015). Pharmacological activation of myosin II paralogs to correct cell mechanics defects. Proc Natl Acad Sci U S A 112, 1428–1433.

2. The Shh expression domain appears smaller as well as visually reduced upon MyoII inhibitor (blebistatin) treatment (Supplementary Figure 1d, e). The Shh expression domain also appears

to be smaller (present in fewer cells) in the conditional knockouts (Supplementary Figure 1m,n) and there is a corresponding reduction in Ptch1 expression (Supplementary Figure m,n). My interpretation is that this suggests that instead of being upstream as proposed, Shh might be either downstream of MyoII (by downstream, this might be quite indirect based on the prolonged time period when Myosin is inhibited/lost). If there is a reduction in Hedgehog pathway strength upon Myosin inhibition, it calls into question the proposed mechanism where Shh acts upstream of Myosin activation.

Response: During normal incisor development, Shh expression is initiated in two distinct phases, first in the initiation knot (IK) at the anterior border of the tooth germ (S4g Fig), and later in the enamel knot (EK) between the two epithelial extensions (S4k Fig). Shh expression in the IK gradually disappears after E13.0, just as its expression in the EK begins to emerge at E13.5 (S4i,k Fig) (Du et al., 2017). In our whole mandible explant culture system, incisor germs cultured for 48 hours happen to fall within the IK-EK transition period, and therefore samples often have varied *Shh* expression. This contributed to the seemingly low Shh expression seen in the blebbistatin-treated sample image. To study whether blebbistatin downregulates *Shh* expression in the EK, we have now performed new experiments and cultured whole mandibles for 60 hours, at which point the EK is fully formed. We also opted for RNAscope, instead of the traditional section in situ, to detect *Shh* mRNA, due to RNAscope's high sensitivity. Our results showed that blebbistatin-treated EK continued to maintain robust *Shh* expression (S1e-g Fig), although the dental cord was wider and the invagination was reduced, as expected for MyoII-inhibited samples.

To concretely determine how *Shh* expression and response to Shh might change in the absence of MyoII activity, we turned to our MyoII genetic mutants and performed *Shh* and *Ptch1* RNAscope across different timepoints from E12.5 to E14.5. We quantified the number of *Shh*-positive cells and the number of *Ptch1* puncta per cell, and these data are now presented in S4g-p Fig. No statistically significant differences were observed between control and mutant samples at these stages, suggesting that MyoII is not required for Shh signaling. We do however agree with the reviewer that there is perhaps a slight reduction of Shh signaling at E14.5, although not significant. We have therefore left open this possibility and added the following sentence in the Discussion on page 20 of the revised manuscript with tracked changes: "*However, as there is a slight, although not statistically significant, reduction in Shh signaling from the Myh9/10$^{epi\text{-}cko}$ enamel knot at 14.5 (S4o,p Fig), it remains possible that MyoII can modulate or propagate Shh signaling at later stages*." But given that the cell adhesion and invagination phenotypes took place in the MyoII mutants when Shh signaling was still clearly intact at E12.5-E13.0, the most logical conclusion is that Shh acts upstream of MyoII to regulate these processes during early tooth development.

Lastly, we would like to provide the rationale for choosing our experimental timepoints, as the reviewer has questioned the prolonged myosin loss. For mouse genetic experiments, because it takes time for gene deletion and protein turnover to take place, it is typical to wait for 24-48 hours after tamoxifen administration before phenotypic analyses are conducted. In the case of K14CreER, it becomes activated at E11.5, and we have chosen to perform most of our analyses

at E13.0, because this is when most myosin IIA and IIB are turned over and before structural phenotypes appear (S2 Fig). We have also looked at tooth morphologies at later stages because the time scales for these phenotypes to emerge is inherently longer than cell biological phenotypes. Similarly, for the blebbistatin experiments, because our goal was to observe tissue morphological changes following growth, we have allowed the tooth to develop for 48-60 hours before harvesting. These phenotypes are by no means a "direct" downstream effect of blebbistatin, as the reviewer has pointed out, but nonetheless a consequence of altered cell adhesion and movement, as we subsequently showed using mouse mutants.

1. Du, W., Hu, J. K., Du, W. and Klein, O. D. (2017). Lineage tracing of epithelial cells in developing teeth reveals two strategies for building signaling centers. J. Biol. Chem.

3. There is an increase in apoptosis (Supplemental Figure 1G,H) that occurs where the cyst forms (and where Shh is normally expressed). The role of apoptosis is dismissed in the results because they previously found that other mutants with apoptosis such as K14CreER;Piezo1 still invaginate normally (Supplemental Figure 3C). However, upon close inspection, the domain of apoptosis is different upon Piezo1 conditional deletion compared to MyoII deletion, which makes this evidence for dismissing the role of apoptosis in the phenotype unconvincing. Is it possible that a particular domain of cells is being lost and then replaced with other cells that then are responsible for the phenotype?

Response: We thank the reviewer for raising this question. Our reasoning was that because there was a higher amount of cell death occurring in the *Piezo1* mutant tooth placode, and yet they continued to undergo normal morphogenesis, it is unlikely that apoptosis alone would result in the phenotype that we observed in the MyoII mutants. In addition, given that we did not observe increased apoptosis in the MyoII mutants until E13.5, the cell adhesion and migration phenotypes at E13.0 would not have been caused by cell death. However, it is possible that cell death contributed to the cyst formation after E13.5, and later stage development can be affected as a result. Reviewer #1 had a similar question (please see our response to their point #2 above), and we have now added a discussion on this possibility in the revised manuscript.

Minor Concerns:
-Related to major point #1, cyclopamine is not as specific of a HH inhibitor as more modern drugs like SANT. Genetic approaches (such as Smo conditional knockouts) are feasible and would be more compelling.

Response: Testing the epithelium-specific response to Hh signaling through Smo conditional deletion will be an important experiment that we plan to do in the future. Generating these mutants is unfortunately outside the timeframe of the revision period. As cyclopamine is a widely

used and acceptable inhibitor of Hh signaling, we believe that results from our cyclopamine studies remain compelling.

-Page 6: Myosin IIA and IIB double knockouts were performed because the single mutants did not produce phenotypes (data not shown). Unsubstantiated statements like this are not particularly helpful. Please either show the data in a supplemental figure or remove this statement completely.

Response: We apologize for the oversight and have now removed this sentence.

-Supplementary Figure 2O,P. While I agree that MyoIIA is substantially reduced in the mesenchyme, there still appears to be a significant amount of MyoIIB in the Msx1CreER-driven mesenchymal deletion. If this is still present at this level, it is harder to conclude that mesenchymal MyoII is not required for proper epithelial invagination.

Response: We completely agree with the reviewer that it would not be accurate to conclude that mesenchymal MyoII is not required for epithelial invagination when residual IIB proteins were still observed in some cells. Because the perdured MyoII expression was also seen in our epithelial MyoII mutants, which did develop a phenotype, MyoII likely plays a more dominant and tissue-autonomous role in the epithelium. We have therefore modified the relevant section (page 9 of the revised manuscript with tracked changes) with the following sentence: "*While this result may be due to perdured IIB expression in some mesenchymal cells (S2p Fig), comparisons of the Myh9/10$^{epi\text{-}cko}$ and Myh9/10$^{mes\text{-}cko}$ mutants indicate that the tissue-autonomous function of epithelial MyoII is critical for proper tooth formation*".

Reviewer #3:

Overall: Now the field has learned that diffusible morphogens are important for organ morphogenesis, yet how they are regulated to be expressed in certain cell types, locations and how they affect down stream cellular events to mediate phenotype is a fundamental question the field faces. This study demonstrate Shh- Non-myosin IIA and IIB axis using incisor epithelial invagination as the example. Specifically, they characterized the role of Non-myosin IIA and IIB during incisor epithelial invagination via cell adhesion and movement. The quality of data is high and the text is well written. While the experimental approaches are reasonable as a whole, some missing gaps – especially on the regulatory role of Shh and invaginating suprabasal epithelial cells - still exist. Nevertheless, this work should be of PLoS Genetic standard after adequate revision.

Strength:
- the use of tissue specific conditional knockout transgenic mice.
- Good cell migration analysis Identification of key group of suprabasal epithelial cells responsible for driving NM-mediated invagination

Response: We would like to first thank the reviewer for recognizing the quality of our data and the manuscript's suitability for PLOS Genetics. We also appreciate the reviewer's feedback that the manuscript would benefit by gaining further understanding of Shh's regulatory role and the mechanism enabling spatially specific cell movement. To that end, we have added substantial data to demonstrate that Shh signals through PI3K/AKT to activate MyoII in the dental epithelium and to promote convergent movement during placode invagination (please see our response to Reviewer #1's points #4 and #5). However, as AKT activity is not unique to suprabasal cells, how specificity is achieved remains an important question that we plan to investigate in the future. Our results here unveiled how morphogens can activate cells' mechanical machineries to power the invagination of a stratified epithelium, and building the tools needed to research the spatiotemporal regulation of this process will be an important next step.

Comments
1. NMIIs are integral molecules of cell migration – hence it is reasonable that knocking down of NMII affects epithelial invagination. Nevertheless, according to Fig 5m, cell velocity did not change significantly between control and myo9/10 knockout cells – the significant difference lies in efficiency and speed of turning angle – reflecting that NMII knockdown actually affected directed collective cell migration. Is this affected by cell adhesion? The authors should perform small molecule treatment targeting cell adhesions to show the importance of cell adhesion during collective migration in incisor invagination.

Response: We thank the reviewer for pointing out the potential requirement of cell-cell adhesion in directing the convergent movement and dental epithelial invagination. We have previously generated a triple mutant with conditional epithelial deletion of αE-catenin, YAP, and TAZ, where YAP/TAZ deletion rescued the cell differentiation phenotype caused by loss of αE-catenin, but not its cell-cell adhesion phenotype (Li et al., 2016). In these mutants, small holes were readily detected throughout the epithelium, yet the dental cord was able to narrow and the tooth underwent normal invagination. Similarly, deletion of the small Rho GTPase Cdc42 in the molar epithelium also disrupted cell-cell adhesion, but convergent extension and invagination appeared to be normal (Zheng et al., 2018). We unfortunately don't have access to these mutants currently to perform live imaging and assess cell movement. But based on the lack of phenotypes associated with dental cord narrowing and epithelial invagination in these mutants, our interpretation is that sufficient convergent movement could still take place to promote invagination when certain cell-cell adhesion, e.g. at adherence junctions, is disrupted. Blocking E-cadherin antibody has been previously used to inhibit E-cadherin-mediated adhesion in cultured cell monolayers. However, given the results above and our experience that live dental

epithelium is fairly impenetrable to large molecules, the use of blocking antibody is unlikely to work.

In the developing dental epithelium, cell-cell adhesion is likely mediated by not only adherence junctions, but also tight junctions and desmosomes, as components of these adhesion complexes are expressed in the suprabasal cells (Ye et al., 2022). Because both tight junctions and desmosomes are also mechanosensitive (Schwayer et al., 2019; Sadhanasatish et al., 2023), force transmissions through these adhesion complexes may help explain why the αE-catenin/YAP/TAZ triple mutant incisors could still invaginate. Unfortunately, there are no good small molecule inhibitors for disrupting these complexes and to specifically test which of these adhesions is important for directing the convergent movement. We have therefore attempted to disrupt all adhesions by treating tissues with 4mM EGTA (Gonzalez-Mariscal et al., 1990; Kimura et al., 2012). However, this caused significant dissociation between cells and tissue destruction during imaging (please see image below). Therefore, we could not reliably track and quantify cells, and we did not include this result in the manuscript.

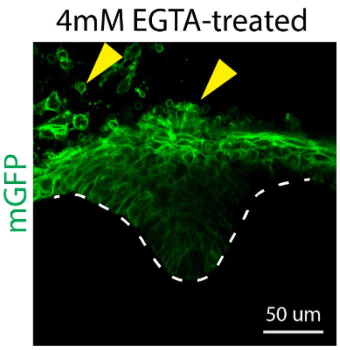

So while we believe that loss of cell-cell adhesion contributed to the MyoII cell movement phenotype, we don't currently have the tools to specifically interrogate how adhesion controls the process. These aspects were carefully discussed in the discussion. The role of cell-cell adhesion deserves a thorough analysis using mouse genetics, and we apologize that we couldn't fully address this point here. We hope this reviewer would agree that such an analysis is outside the timeframe and scope of this revision, and it won't alter our results or conclusions in this paper. We are excited to continue this investigation in the future.

1. Gonzalez-Mariscal, L., Contreras, R. G., Bolívar, J. J., Ponce, A., Chávez De Ramirez, B. and Cereijido, M. (1990). Role of calcium in tight junction formation between epithelial cells. Am J Physiol 259, C978-986.
2. Kimura, T. E., Merritt, A. J., Lock, F. R., Eckert, J. J., Fleming, T. P. and Garrod, D. R. (2012). Desmosomal adhesiveness is developmentally regulated in the mouse embryo and modulated during trophectoderm migration. Developmental Biology 369, 286–297.
3. Li, C.-Y., Hu, J. K., Lu, H., Lan, J., Du, W., Galicia, N. and Klein, O. D. (2016). αE-catenin inhibits YAP/TAZ activity to regulate signalling centre formation during tooth development. Nat Commun 7, 12133.

4. Sadhanasatish, T., Augustin, K., Windgasse, L., Chrostek-Grashoff, A., Rief, M. and Grashoff, C. (2023). A molecular optomechanics approach reveals functional relevance of force transduction across talin and desmoplakin. Science Advances 9, eadg3347.
5. Schwayer, C., Shamipour, S., Pranjic-Ferscha, K., Schauer, A., Balda, M., Tada, M., Matter, K. and Heisenberg, C.-P. (2019). Mechanosensation of Tight Junctions Depends on ZO-1 Phase Separation and Flow. Cell 179, 937-952.e18.
6. Ye, Q., Bhojwani, A. and Hu, J. K. (2022). Understanding the development of oral epithelial organs through single cell transcriptomic analysis. Development 149, dev200539.
7. Zheng, J., Nie, X., He, L., Yoon, A. J., Wu, L., Zhang, X., Vats, M., Schiff, M. D., Xiang, L., Tian, Z., et al. (2018). Epithelial Cdc42 Deletion Induced Enamel Organ Defects and Cystogenesis. J Dent Res 97, 1346–1354.

2. Most of the functional tests were carried out at E13.5, while the most drastic morphological changes caused by Myh9/10 knockout actually occur on E14.5. Some of the key experiments (e.g., Shh, cyclopamine, calyculin A rescue, vector field) should be carried out for E14.5 to show its robustness.

Response: We thank the reviewer for suggesting this experiment. We performed most of our MyoII mutant characterization using E13.0 embryos because this was soon after most of the myosin heavy chains IIA and IIB were removed but before there were clear invagination defects and significant tissue structural damages that could confound the analyses. In doing so, we hope to capture the more immediate effect of MyoII disruption, and to avoid attributing the more downstream changes to MyoII functions, which were a concern of Reviewer #2.

For explant studies, we have used E12.5 embryos because any perturbation needs to take place before dental cord narrowing. However, it would indeed be very interesting to test the effect of cyclopamine and calyculin A on E13.5 incisors, which are starting to narrow at the neck region and may produce a visually more distinct phenotype, as the reviewer suggested. We have therefore carried out this experiment using E13.5 mandibular tissue slices, as whole mandibles from this stage are much bigger than E12.5 mandibles and could not be easily maintained in culture. After culturing for 24 hours, we obtained the same result as before. We found that cyclopamine treatment prevented dental cord narrowing and reduced invagination, but calyculin A was able to rescue these phenotypes. Representative images and quantifications are now included in S10 Fig. We did not repeat the experiment by culturing E14.5 incisors, as their dental cords are already narrowed.

3 .The most interesting but overlooked finding of this study is actually the specific cells involved – the Lamin A/C+ suprabasal cells – this is actually just a small population of cells amongst all the NM+ epithelial cells. How are they activated? The authors targeted Shh and used Ptch1

expression, Cyclopamine, and Calyculin A rescue experiments to demonstrate that Shh could be an upstream regulator of NMII. However, the fact that Shh expression is only localized at the initiation knot (IK) and Ptch1 is homogeneously expressed in both epithelium and mesenchyme, reflect that the activation of these Lamin A/C+ suprabasal cells could be controlled by a different and more spatially precise mechanism. It is a good opportunity to dig deeper and make this manuscript even more valuable. Authors can do their best in this front.

Response: We too found it very interesting how Shh signaling leads to convergent cell movement primarily in the upper suprabasal cells. As the reviewer pointed out, given that *Ptch1* expression and pMLC expression are present in most cells, activation of actomyosin tension alone is not sufficient to regulate this process. Our hypothesis is that actomyosin function is utilized differently in different cells and in different parts of the cells, resulting in spatiotemporally polarized adhesion and movement in the suprabasal layer. We showed that Shh plays an important role in activating MyoII activity in general but other signaling regulations are likely in place to coordinate the spatiotemporal functions of actomyosin to produce distinct cell behaviors. Observing actin dynamics and myosin activity in live incisors, identifying a suprabasal-specific Cre to interrogate the function of other factors, and delineating the roles between adhesion and movement will help elucidate this very interesting question. These are however outside the scope of this current study, and we hope to pursue this further in our future investigation!
